# Roles and Responsibilities of the Global Specialist Digital Health Workforce: Analysis of Global Census Data

**DOI:** 10.2196/54137

**Published:** 2024-07-25

**Authors:** Kerryn Butler-Henderson, Kathleen Gray, Salma Arabi

**Affiliations:** 1 School of Nursing, Paramedicine, and Healthcare Sciences, Charles Sturt University, Locked Bag 588, Wagga Wagga, New South Wales, 267861 0263384480, Australia; 2 Digital Health Hub, College of STEM, RMIT University, Bundoora, Australia; 3 Centre for Digital Transformation of Health, University of Melbourne, Parkville, Australia

**Keywords:** workforce, functions, digital health, census, census data, workforce survey, survey, support, development, use, management, health data, health information, health knowledge, health technology, Australia, New Zealand, online content, digital data

## Abstract

**Background:**

The Global Specialist Digital Health Workforce Census is the largest workforce survey of the specialist roles that support the development, use, management, and governance of health data, health information, health knowledge, and health technology.

**Objective:**

This paper aims to present an analysis of the roles and functions reported by respondents in the 2023 census.

**Methods:**

The 2023 census was deployed using Qualtrics and was open from July 1 to August 13, 2023. A broad definition was provided to guide respondents about who is in the specialist digital health workforce. Anyone who self-identifies as being part of this workforce could undertake the survey. The data was analyzed using descriptive statistical analysis and thematic analysis of the functions respondents reported in their roles.

**Results:**

A total of 1103 respondents completed the census, with data reported about their demographic information and their roles. The majority of respondents lived in Australia (n=870, 78.9%) or New Zealand (n=130, 11.8%), with most (n=620, 56.3%) aged 35‐54 years and identifying as female (n=720, 65.3%). The top four occupational specialties were health informatics (n=179, 20.2%), health information management (n=175, 19.8%), health information technology (n=128, 14.4%), and health librarianship (n=104, 11.7%). Nearly all (n=797, 90%) participants identified as a manager or professional. Less than half (430/1019, 42.2%) had a formal qualification in a specialist digital health area, and only one-quarter (244/938, 26%) held a credential in a digital health area. While two-thirds (502/763, 65.7%) reported undertaking professional development in the last year, most were self-directed activities, such as seeking information or consuming online content. Work undertaken by specialist digital health workers could be classified as either leadership, functional, occupational, or technological.

**Conclusions:**

Future specialist digital health workforce capability frameworks should include the aspects of leadership, function, occupation, and technology. This largely unqualified workforce is undertaking little formal professional development to upskill them to continue to support the safe delivery and management of health and care through the use of digital data and technology.

## Introduction

The importance of a specialist digital health workforce to support the development, use, management, and governance of health data, health information, health knowledge, and health technology has been well-documented [1], particularly through the transformation of digital health during the COVID-19 pandemic. This largely hidden workforce [2] supports the digital health needs for care delivery and management. They are the clinical coders, health informaticians, health information managers, health librarians, health technologists, and so many other occupational specialties who work behind the clinical scenes to ensure that care providers and health managers have the right data, information, and knowledge at the right time and right place [1]. However, there is a lack of accurate data about this specialist digital health workforce to understand their educational needs, their roles and functions, and their professional development needs. This gap in evidence creates challenges for workforce and education planning and forecasting.

The Global Digital Health Workforce Census was launched in 2018 following a rigorous development process [3]. The census stemmed from a collaborative effort between the University of Tasmania and the University of Melbourne using a Delphi approach. A 10-member expert panel, comprising representatives from key stakeholders, identified issues during a focus group, forming the basis for a health information workforce minimum data set. The items in the census tool were based on existing workforce data items from other surveys and census data sets, and were initially developed with Australian and New Zealand experts. Based on the Health Workforce Australia Report [4], which called for improved data collection about the workforce, the census was referred to as the Australian Health Information Workforce Census. Following the 2018 census [1], the project undertook a validation study to globalize data items with the 2021 census, a smaller pilot with a more global group of participants [5]. The census was referred to as the Global Health Informatics, Digital, Data, Information, and Knowledge (HIDDIN) Workforce Census. The 2023 census was the first full census with global participants, renamed to the Global Specialist Digital Health Workforce, as defined by Butler-Henderson et al [6]. In addition, the census project team worked with Telstra Health to incorporate their Women and Digital Health [7] survey questions into the census. The purpose of this paper is to present the data from the 2023 census related to the roles and functions of the various specialist occupational groups in the specialist digital health workforce.

## Methods

### Ethical Considerations

The census was held online from July 1 to August 13, 2023. The census project was approved by the RMIT University Human Research Ethics Committee (#26607). No identifiable information is collected in the census and the survey system automatically allocated a unique identifier code to each response. For any questions with <5 responses, the number of responses is not reported to maintain confidentiality. Participants were not compensated for completing the census.

### Survey Instrument

The census is a survey deployed through the Qualtrics survey system at RMIT University. It consists of 186 questions across 9 sections, as outlined in Textbox 1.

Textbox 1. **Census sections and question topics in each section.**

**Demographic**

- Country, state, and postcode of residence
- Country of birth and citizenship status
- Year of birth
- Gender
- Indigenous or ethnic group
- Disability

**Professional membership and health practitioner registration**

- What digital health memberships they hold
- If they are a registered health professional and field
- Hours worked in clinical role

**Formal education**

- Specialist digital health formal education at vocational or higher education level
- Clinical qualifications
- Other relevant qualifications

**Credentials**

- Relevant credentials

**Occupation and paid employment information**

- Discipline group
- Time worked in the specialist digital health workforce
- Seeking work
- Current digital health role(s)—for up to two roles, including country, state, postcode, role title, time in role, role intentions next 12 months, top 5 functions, permanency, organization type (both public/private and service type, eg, hospital, educational, department, not for profit), and renumeration
- How many different roles they have

**Unpaid and voluntary work**

- Voluntary roles and other unpaid related work

**Professional development**

- What professional development they have done in the last 12 months
- Needs and plans for next three years

**Workforce intentions**

- How much longer they plan to stay in the workforce
- Why they will leave
- If they will continue to volunteer or do unpaid specialist digital health work

**Women and digital health**

- Questions from the Women and Digital Health survey

### Recruitment

The promotion of the census occurred in multiple ways. The 2023 census was supported by the Australian Digital Health Cooperative Research Centre, Australian Department of Health and Aged Care, Telstra Health, Australasian Institute of Digital Health, Australian Library and Information Association Health Libraries Australia, and the Health Information Management Association of Australia, all of which promoted the census to their networks. The census was launched at the 2023 international health and medical informatics conference, MedInfo. It was also promoted through other professional membership organizations, such as the International Federation of Health Information Management Associations and several other national organizations, such as ANDHealth, and through academic organizations. The census was advertised in several different publications, such as Pulse+ IT and What the Health. Several posts were shared on the census LinkedIn channel and X (formerly Twitter) account. Lastly, individuals could register for a distribution list, which received 2 alerts about the census.

Completion of the census was open to those who self-identified as part of the specialist digital health workforce. The following general guidance was provided:

*You are part of the workforce if any part of your role (including volunteer or actively seeking) includes a function (listed below) related to health data, information, or knowledge. You may undertake a role that has both a Specialist Digital Health component and another component (for example, clinical or management). For this Census, only consider the Specialist Digital Health component. Functions could include analysing, designing, developing, implementing, maintaining, managing, operating, evaluating, or governing the data, technology, systems, and services for the health sector. You might not identify as part of the Specialist Digital Health workforce if the primary function of your role is limited to using health data, information, or knowledge but none of the other functions listed above.*

An information sheet was provided so that participants could make an informed decision about participation. At the start of the census, participants were reminded about the information sheet and asked to review the questions with regard to providing consent. If they did not consent to participate in the study, they were taken out of the survey. The census took an average of 14 minutes to complete; however, this varied depending on how much detail the participant chose to provide.

### Statistical Analysis

Once the survey was closed, the data for all responses was cleaned and only responses that completed section 1 were included in the analysis. Most data items were analyzed using descriptive statistics, such as the number and percentage of responses. When there were fewer than 5 responses, the data is presented as “<5.” Only items relevant to capabilities and skills were analyzed for this manuscript, and not all sections of the census are presented in this paper due to the relevance of the topic.

Participants were asked to provide up to 5 functions related to their primary specialist digital health role. All responses were grouped and, using NVivo 14.23 (Lumivero), analyzed for word frequency. The top 5% of the most frequently reported terms were then thematically analyzed, using a modification of the themes identified by Prommegger et al [8]. While Prommegger et al [8] examined occupational aspects, human aspects, and technological aspects, this study examined leadership aspects, functional aspects, occupational aspects, and technological aspects. The top 4 occupational specialties were identified, and the functions listed by respondents who identified with those occupational specialties were then extracted and thematically analyzed in the same fashion, where 5 or more participants identified the term.

## Results

### Overview

Complete responses for all of section 1 were received from 1103 participants. The majority of responses were from Australia (n=870, 78.9%). Countries with more than 5 responses are shown in Table 1. More than half (n=620, 56.3%) of participants were aged 35‐54 years, and two-thirds (n=720, 65.3%) identified as female. A total of 73 (7.1%) participants identified as Indigenous and 42 (3.8%) as living with a disability.

**Table 1.** Participants’ characteristics of the 2023 Global Specialist Digital Health Workforce Census (N=1103).

Characteristic and selections		Participants, n (%)
**Countries (>5 respondents)**		
	Australia	870 (78.9)
	New Zealand	130 (11.8)
	United States	33 (3.0)
	England	9 (0.8)
	Nigeria	8 (0.7)
	Saudi Arabia	6 (0.5)
	Spain	5 (0.5)
	India	5 (0.5)
**Age group (years)**		
	<25	15 (1.4)
	25‐34	123 (11.2)
	35‐44	283 (25.7)
	45‐54	337 (30.6)
	55‐64	273 (24.8)
	≥65	72 (6.5)
**Gender**		
	Female	720 (65.3)
	Male	364 (33.0)
	Nonbinary, gender-fluid, agender	8 (0.7)
	Prefer not to say	11 (1.0)

### Occupational Specialization

Respondents were asked to select which occupational specialty they identified with from a list of 16 occupation areas previously identified through the analysis of responses to the 2018 census. The top four occupational specialties were health informatics (n=179, 20.2%), health information management (n=175, 19.8%), health information technology (n=128, 14.4%), and health librarianship (n=104, 11.7%; Table 2). When asked how they classify their occupation against the major categories used by the Australian Bureau of Statistics (ABS) [9], 90% (n=797) of participants identified as managers or professionals. While these classifications are based on the Australian context, the census recognized the international nature of the digital health workforce. Respondents from other countries were encouraged to align their occupations with the provided categories, acknowledging that the ABS classifications served as a reference point for a standardized comparable analysis across diverse geographical contexts. This approach facilitated a more inclusive representation of the global specialist digital health workforce while maintaining a structured framework for analysis.

Respondents also were asked to review the definition of 8 digital health profiles developed by the Australian Digital Health Agency (ADHA) [10] and to select which one they identified as most aligning with their work. These 8 digital health profiles capture the diverse perspectives of the health workforce based on individual roles in the design, development, implementation, and adoption of digital technologies. The profiles include patient, carer, and consumer; frontline clinical; digital champion; clinical and technology bridging; education and research; technologist; leadership and executive; and business, administration, and clinical support [10]. There is no known analysis of the ADHA profiles previously published. In this 2023 census, there was a distribution across a range of profiles, with the top four being leadership and executive (n=174, 19.6%); education and research (n=162, 18.3%); business, administration, and clinical support (n=159, 17.9%); and clinical and technology bridging (n=136, 15.3%). Only 16.7% (n=148) of respondents identified as either a technologist or digital champion.

Table 2 summarizes respondents’ categorization of their occupations.

**Table 2.** Occupational specializations and classifications in the 2023 Global Specialist Digital Health Workforce Census (n=886).

		Participants, n (%)
**Occupation area**		
	Biomedical engineering	<5
	Clinical coding	47 (5.3)
	Clinical documentation improvement	31 (3.5)
	Epidemiology	5 (0.6)
	Health artificial intelligence	7 (0.8)
	Health cybersecurity	<5
	Health data science/analytics	53 (6.0)
	Health informatics	179 (20.2)
	Health information management	175 (19.8)
	Health information technology	128 (14.4)
	Health innovation	56 (6.3)
	Health interoperability	28 (3.2)
	Health librarianship	104 (11.7)
	Health simulation	<5
	Health technology assessment	7 (0.8)
	Translational bioinformatics	<5
	Unable to classify	54 (6.1)
**Occupation classification**		
	Clerical or administrative worker	35 (4.0)
	Community or personal service worker	<5
	Laborer	<5
	Manager	323 (36.5)
	Professional	474 (53.5)
	Sales worker	<5
	Technician or trades worker	10 (1.1)
	Unable to classify	38 (4.3)
**Australian Digital Health Agency classification**		
	Business, administration, and clinical support	159 (17.9)
	Clinical and technology bridging	136 (15.3)
	Digital champion	54 (6.1)
	Education and research	162 (18.3)
	Frontline clinical	34 (3.8)
	Leadership and executive	174 (19.6)
	Patient, consumer, and carer	27 (3.0)
	Technologist	94 (10.6)
	Unable to classify	45 (5.1)

### Qualifications

Participants were asked about their qualifications. With regard to a qualification in a specialist digital health area, the majority (589/1019, 57.8%) of respondents reported no formal educational qualification in a specialist digital health area. Further, only one-quarter (244/938, 26%) reported any industry-issued credential in a digital health area. Of 1033 responses, 30% (n=310) reported that they were a registered clinician.

With regard to professional development activities undertaken in the last year, 65.7% (502/763) reported undertaking some form of professional development. Participants were given the option to identify where they had undertaken the activity and could select more than one organization that delivered that professional development activity. Self-directed professional development activities, such as information seeking, reading/listening/watching blogs/podcasts/vodcasts, and other self-directed activities, were the most reported (676/2438, 27.7%) form of professional development activity (Table 3).

**Table 3.** Sources of professional development activities reported in the 2023 Global Specialist Digital Health Workforce Census.

Organization delivering activity	Participants, n (%^a^)
Government	223 (9.1)
Industry organization	509 (20.9)
Membership organization	511 (21.0)
Self	676 (27.7)
Training provider	162 (6.6)
Workplace	357 (14.6)

a Percentages are based on the total number of reported professional development activities (N=2438).

### Employment

With regard to their primary specialist digital health role, more than half (n=487, 55%) of respondents reported that they had worked in their current role for <10 years (Table 4). Three-quarters (n=607, 76.5%) of respondents reported that they were in a permanent specialist digital health role. Representing two-thirds (n=544, 68.6%) of respondents, the top four organizational types were hospital (n=300, 37.8%), health technology organization (n=96, 12.1%), state health department (n=83, 10.5%), and educational facility (n=65, 8.2%). Most (n=552, 69.6%) were public organizations.

**Table 4.** Employment characteristics of primary specialist digital health role in the 2023 Global Specialist Digital Health Workforce Census.

Characteristic and selections		Participants, n (%)
**Years in current role (n=886)**		
	<5	244 (27.5)
	5‐9	184 (20.8)
	10‐14	142 (16.0)
	15‐19	84 (9.5)
	20‐24	87 (9.8)
	≥25	145 (16.4)
**Employment status (n=793)**		
	Casual	28 (3.5)
	Contract	142 (17.9)
	Permanent	607 (76.5)
	Self-employed	16 (2.0)
**Employment setting (n=793)**		
	Community health care service	25 (3.2)
	Defense force/military	<5
	Educational facility	65 (8.2)
	Federal health organization	44 (5.5)
	Health technology organization	96 (12.1)
	Hospital	300 (37.8)
	Indigenous health service	9 (1.1)
	Local health service/district/network	57 (7.2)
	Other not-for-profit organization	29 (3.7)
	Other private organization	32 (4.0)
	Other public/government organization	26 (3.3)
	Primary care or primary health network	16 (2.0)
	Private practice	6 (0.8)
	Residential health care facility	<5
	State health department	83 (10.5)
**Employer status (n=793)**		
	Not-for-profit	73 (9.2)
	Private	147 (18.5)
	Public	552 (69.6)
	Public/private partnership	21 (2.6)

### Functions

The Census asked respondents to list the top five functions of their primary specialist digital health role; 792 respondents provided between one to five functions. Thematic analysis of these functions (as described in the Methods section using a modified list of themes [8]) identified four broad ways of describing their work responsibilities, with example terms shown in Textbox 2.

1. Leadership aspects: these are functions related to leadership.
2. Functional aspects: these are functions related to the operational aspects of roles.
3. Occupational aspects: these are functions that describe the occupation.
4. Technological aspects: these are functions related to the technological aspects of the occupation.

The analysis identified that there was a broad range of functions across these themes, which is to be expected when analyzing the functions across 4 occupational specialist groups representing more than half of the workforce. There was a total of 1353 functions provided across these 4 groups. The functions of health informatics (n=183 responses for functions), health information management (n=175), health information technology (n=135), and health librarian (n=104) were themed.

Textbox 2. **Example terms for describing work responsibilities in the 2023 Global Specialist Digital Health Workforce Census.**

**Leadership aspects**

Leadership, policy, strategic, strategy

**Functional aspects**

Advice, analysis, governance, manage, searching, teaching

**Occupational aspects**

Design, development, plans, research, support, service

**Technological aspects**

Applications, data, digital, software, systems, user

## Discussion

Traditionally, throughout the world, capability and competency frameworks have been developed by experts based on their many years of experience. Thus the existing frameworks for digital health specialist occupational areas in many countries, including but not limited to those shown in Table 5, have been developed by industry and academic experts. However, it is crucial to acknowledge the limitation of our findings, as nearly 80% of responses came from Australia. This geographic concentration may limit the generalizability of the results, particularly for countries with single-digit responses. We recognize that while the census provides valuable insights, its predominantly Australian data set may impact the applicability of our conclusion globally. Therefore, it is imperative to interpret our framework recommendations within the context of this geographic bias. Nevertheless, this approach was once the only way to develop these frameworks; today, we have access to a large resource of data about the workforce to inform these frameworks. The Global Specialist Digital Health Workforce Census is one such source.

This paper shows how capability frameworks can be informed by data from those working in these roles. The insights from this analysis not only inform the types of roles and their functions and responsibilities but also help validate expert-originated frameworks and identify new emerging roles with the analysis of census data over time. The four themes identified in this review, leadership aspects, functional aspects, occupational aspects, and technological aspects, and associated functions within each theme, could guide future capability framework development for the specialist digital health workforce (Textbox 3).

**Table 5.** Distribution of work responsibilities by occupational group and theme in the 2023 Global Specialist Digital Health Workforce Census.

Occupational specialist	Responses, n	Functions listed, n	Functions included in theme analysis, n	Leadership aspects, n (%)	Functional aspects, n (%)	Occupational aspects, n (%)	Technological aspects, n (%)
Health informatics	183	584	141	7 (5.34)	44 (33.59)	39 (29.77)	41 (31.30)
Health information management	175	610	127	6 (4.88)	44 (35.77)	36 (29.27)	37 (30.08)
Health information technology	135	477	85	4 (4.88)	20 (24.39)	30 (36.59)	28 (34.15)
Health librarian	104	432	106	6 (5.88)	33 (32.35)	26 (25.49)	37 (36.27)

Textbox 3. **Modified, with addenda, list of competency lists in specialist digital health occupational areas [11].**

**Health data scientist**

- Canadian Institute of Health Information [12]

**Health Informatics**

- American Medical Informatics Association [13]
- Australasian Institute of Digital Health [14]
- Digital Health Canada [15]
- Gulf Cooperation Council Health Informatics Workforce Working Group [16]
- Faculty of Informatics United Kingdom [17]

**Health information and communications technologists**

- Health Information Technology Competencies (HITCOMP) [18]

**Health information managers**

- American Health Information Management Association [19]
- Canadian Health Information Management Association [20]
- Global Health Workforce Council [21]
- Health Information Management Association of Australia [22]

**Health librarianship**

- Australian Library & Information Association (Health Libraries Australia) [23]
- Medical Library Association [24]

Of critical concern, this census identified that the broad specialist digital health workforce is largely untrained in digital health capabilities, with more than half (589/1019, 57.8%) reporting that they did not have a specialist digital health qualification. Further, this workforce is not developing these skills consistently through a credentialing program (only 26% hold a credential) or through professional development activities (65.7% reported undertaking professional development in digital health in the past year).

While it could be assumed that most respondents were developing these skills on the job, most (55%) have only been in their role for <10 years, and one-quarter (27.5%) have been in their role for <5 years. On-the-job training is an important factor in improving the quality of health care [25], and the time it takes to become fully productive in a new job is significantly longer in the health workforce, varying depending on the complexity of the job, the individual’s prior experience and skills, and the organization’s orientation and induction process. While the first 90 days are important, it can take years for a new recruit to a role to be fully productive [26].

There is unquestioned recognition that qualifications to practice and continue professional development are critical for safe health care [27]. Yet amid the ever-increasing digital transformation of the health and care sector, this census shows that professional training and continuing professional development of digital health specialists is at least underreported or at worst absent [28-30].

This is the largest known analysis of the functions of the specialist digital health workforce; however, it is acknowledged that this analysis is of 792 respondents and is largely an Australian data set. It is important to note that the recruitment process may introduce response bias, as those who chose to participate may differ systematically from those who did not. The Australian-centric focus of this data set could limit the generalizability of findings to a broader global context. Future censuses, with a more diverse and extensive respondent pool, will be essential to mitigate potential biases and enhance the robustness and representativeness of the analysis.

The specialist digital health workforce has dedicated roles where their primary function is to support the development, use, management, and destruction of health data, health information, health knowledge, and health technology. The Global Specialist Digital Health Workforce Census is the only survey of its kind to capture critical information about this workforce, including the functions and the capabilities required for them to undertake their roles. However, to enhance the depth of this work, it is essential to provide greater granularity about the specific functions these roles entail. Understanding the intricacies of their daily tasks and responsibilities is crucial for a more comprehensive analysis. This overview emphasizes the largely unqualified nature of the workforce and their limited engagement in formal professional development. This underscores the need for a detailed exploration of the functions performed by these roles, which will not only shed light on the current state but also inform the creation of a more nuanced and informed capability framework. Future frameworks should encompass leadership, function, occupation, and technology aspects to offer a holistic perspective on the specialist digital health workforce.

## Abbreviations

ABS: Australian Bureau of Statistics
ADHA: Australian Digital Health Agency
HIDDIN: Health Informatics, Digital, Data, Information, and Knowledge
